# Efficient facemask decontamination via forced ozone convection

**DOI:** 10.1038/s41598-021-91735-w

**Published:** 2021-06-10

**Authors:** Joseph Schwan, Troy R. Alva, Giorgio Nava, Carla Berrospe Rodriguez, Zachary Spencer Dunn, Justin W. Chartron, Joshua Morgan, Pin Wang, Lorenzo Mangolini

**Affiliations:** 1 Department of Mechanical Engineering, University of California Riverside, 900 University Ave, Riverside, CA 92521 USA; 2 Material Science and Engineering Program, University of California Riverside, 900 University Ave, Riverside, CA 92521 USA; 3 Department of Bioengineering, University of California Riverside, 900 University Ave, Riverside, CA 92521 USA; 4 Mork Family Department of Chemical Engineering and Materials Science, University of Southern California, 3650 McClintock Ave, Los Angeles, CA 90089 USA; 5 Protabit LLC, 1010 E Union St Suite 110, Pasadena, CA 91106 USA; 6 Department of Biomedical Engineering, University of Southern California, 1042 Downey Way, Los Angeles, CA 90089 USA; 7 Department of Pharmacology and Pharmaceutical Sciences, University of Southern California, 1985 Zonal Avenue, Los Angeles, CA 90089 USA

**Keywords:** Process chemistry, Public health

## Abstract

The COVID-19 crisis has taken a significant toll on human life and the global economy since its start in early 2020. Healthcare professionals have been particularly vulnerable because of the unprecedented shortage of Facepiece Respirators (FPRs), which act as fundamental tools to protect the medical staff treating the coronavirus patients. In addition, many FPRs are designed to be disposable single-use devices, creating an issue related to the generation of large quantities of non-biodegradable waste. In this contribution, we describe a plasma-based decontamination technique designed to circumvent the shortages of FPRs and alleviate the environmental problems posed by waste generation. The system utilizes a Dielectric Barrier Discharge (DBD) to generate ozone and feed it through the fibers of the FPRs. The flow-through configuration is different than canonical ozone-based sterilization methods, in which the equipment is placed in a sealed ozone-containing enclosure without any flow through the mask polymer fibers. We demonstrate the rapid decontamination of surgical masks using *Escherichia coli* (*E. coli*) and *Vesicular Stomatitis Virus* (VSV) as model pathogens, with the flow-through configuration providing a drastic reduction in sterilization time compared to the canonical approach. We also demonstrate that there is no deterioration in mask structure or filtration efficiency resulting from sterilization. Finally, we show that this decontamination approach can be implemented using readily available tools, such as a plastic box, a glass tube, few 3D printed components, and the high-voltage power supply from a plasma globe toy. The prototype assembled for this study is portable and affordable, with effectiveness comparable to that of larger and more expensive equipment.

## Introduction

In the beginning of 2020, COVID-19 rapidly emerged as a global pandemic that has resulted in hundreds of thousands of deaths. Unprepared for this crisis, healthcare professionals experienced a shortage of disposable Personal Protective Equipment (PPE); in particular Facepiece Respirators (FPRs), such as designated N95 masks in the US and the FFP3 respirators in Europe. These respirators are fundamental tools that protect medical personnel caring for COVID-19 patients. Their designations are earned by the ability to filter out 95% and 99% of particulate matter at or above 0.3 microns in size^1^, the scale of an average virion. The response to this disease has been severely compromised by the lack of adequate PPE. Disruptions to the PPE global supply chain have led to month-long delivery times and massive price increases, leaving doctors and nurses unprotected. As manufacturers are called upon to meet demand, healthcare providers have improvised with less effective substitutes^2^. While, based on manufacturer recommendations, the FPRs are single-use PPE and the US Centers for Disease Control and Prevention (CDC) does not formally recommend their decontamination and re-use, it is acknowledged that in these times of scarcity, decontamination might be considered as a good “practical” solution^3^. The development of standardized approaches to decontaminate fibers, restore filtering electrostatic charge, and in general re-use FPRs is necessary to mitigate impact on both humans and the environment due to their future increased use, as the World Health Organization estimated that a 40% increase of the global PPE supplies will be needed^4^. Existing sterilization methods have been proposed and even adopted in some capacity, though each method appears to have a set of drawbacks or caveats. For example, methods such as autoclaving (steam treatment) and liquid hydrogen peroxide (H_2_O_2_) saturation tend to deform or destroy the mask^5,6^. Similarly, use of UV irradiation has problems with standardization (wavelength, intensity, etc.) and the pathogen protecting effect of shadows^5^. One promising technique is use of gaseous disinfectant species with current work focusing on H_2_O_2_ vapor as it has been proven to work, though it is costly and not a particularly rapid process^5^. Meanwhile, plasma reactors operating in air generate significant amounts of reactive gaseous species such as ozone (O_3_) and H_2_O_2_ with minimal heating^7^.

Ozone (O_3_) is an allotropic form of oxygen with proven pan-viricidal and bactericidal capabilities. It is already widely employed on an industrial scale for wastewater treatment^8^. Its method of sterilization is due to the high reactivity of O_3_ (half-life of 22 min in room temperature), whereby after a collision it is likely to cause oxidation an organic material with the emission of an O_2_ molecule^6,9^. Notably, O_3_ has been reported as effective in de-activating other members of the coronavirus family^10,11^ and the bacteriophage MS2^9^, a virus previously shown to be more resistant to UV-based disinfection with respect to coronaviruses^12^. Additionally, O_3_ can be directly produced from air (e.g. via plasmas or irradiation with UV light) and reconverted into non-hazardous O_2_ with the aid of catalytic converters^13^. Therefore, unlike other compounds, O_3_ can be readily manufactured with cost-effective approaches at the point-of-use. As a gaseous sterilization agent it is a particularly promising option for disinfecting poorly accessible spaces within porous materials, such as FRPs. While both consumer-grade and large-scale O_3_ sterilization devices are widely available for deodorizing and sanitizing both rooms and objects, the design of these systems is not optimized for the disinfection of FPRs. In consumer grade O_3_ sterilization devices objects are loaded into a sterilization chamber which is then sealed and flooded with O_3_. O_3_ passively diffuses into the objects and may slowly enter the porous media of an FPR^9^.

With this contribution we propose an efficient and low-cost O_3_ disinfection approach specifically designed for FPRs. Compressed air is fed into a cylindrical atmospheric pressure Dielectric Barrier Discharge (DBD) plasma that rapidly produces O_3_. The ozone-rich gas flow is then forced through the porous media of the FPR, which is directly connected to the plasma reactor. This method uses a low temperature plasma to produce O_3_, thus avoiding thermal degradation of FPRs as the output gas is near room temperature. The efficacy of this method is compared to the canonical method by quantifying the decontamination effectiveness of surgical masks saturated with either *E. coli* or *Vesicular Stomatitis Virus*. These pathogens were chosen due to their safety, availability, and ease of use. Observations on the structure and filtration efficiency of masks post processing are also taken to understand whether this method of decontamination is non-destructive. Finally, we demonstrate that this approach can be readily adapted as a low-cost solution by using the power supply of a widely available commercial plasma globe toy, a few 3D printed parts, some steel mesh, and a plastic box, to construct a portable low-power system capable of attaining similar disinfection efficiencies.

## Experimental section

### DBD plasma reactor for FPR decontamination

**Figure 1:**
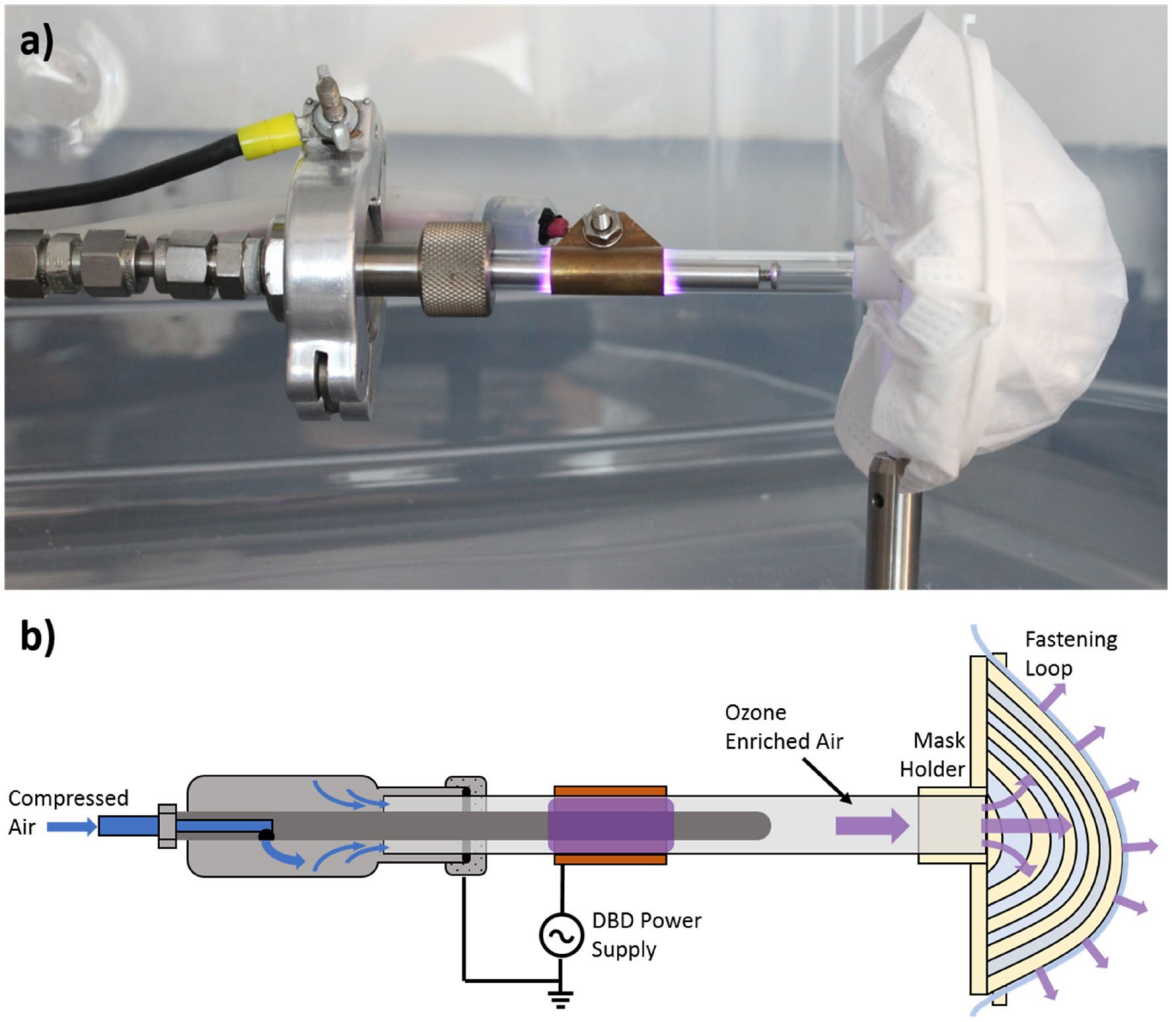
(**a**) Picture and (**b**) schematic of the DBD reactor used in the mask sterilizaton experiments.

Figure 1 shows the DBD reactor used for the mask decontamination experiments. The system comprises a quartz tube (10 mm outer diameter; quartz wall thickness 1 mm), an outer copper electrode connected to a DC power supply (Trek High Voltage Amplifier 10/40A/HS connected to a signal generator; 10 kHz sinusoidal wave with amplitude between 1 and 10 kV) and a 6 mm stainless steel tube as a grounded electrode. Compressed air is flown through the system at constant rate of 10 slm controlled by a King Instruments flowmeter.

The waveform of the discharge voltage *V* was measured from the output of the power supply while the waveform of the discharge charge *Q* was recorded using a 20 nF capacitor, serially connected to the grounded electrode. Both *V* and *Q* were recorded using a digital oscilloscope (Tektronix AFG320).

### Low-cost plasma reactor for FPR decontamination

**Figure 2:**
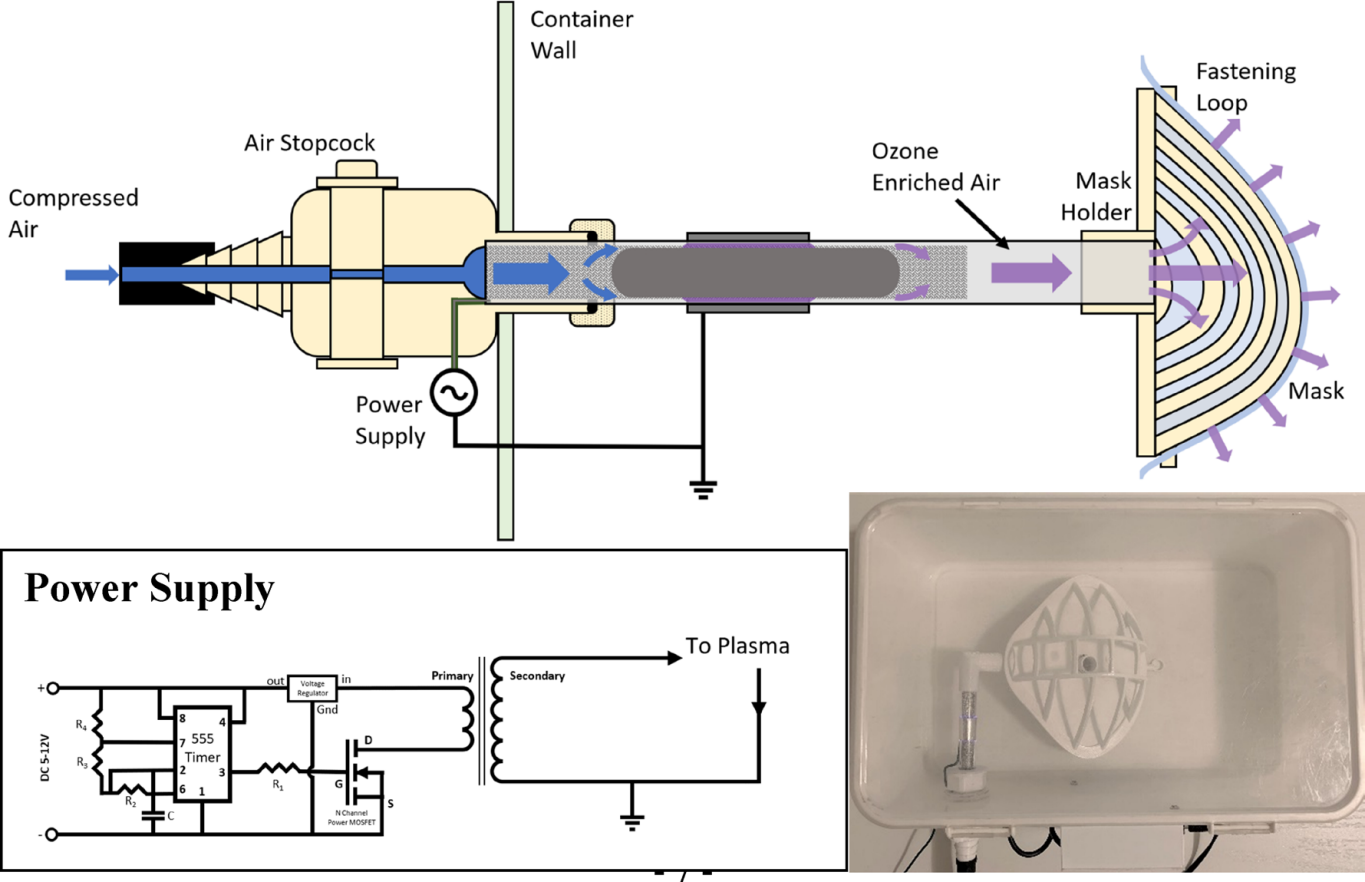
Schematic of cost-effective plasma reactor build by using a commercial toy plasma-ball (top), its simplified circuit diagram (bottom left), and a picture of the system (bottom right).

The power supply from a $25 plasma globe toy (in these experiments a 6-inch Theefun plasma globe) can be used to drive a low-temperature plasma in air to evolve O_3_. This is due to the flyback transformer and the timer circuit within the system that produces low-current and high-voltage (1–6 kV) sawtooth or ramp output signals near or at a frequency of 30 kHz. In order to take advantage of this system, the electrode geometry was modified so that a steel mesh inside the reactor was powered and the external electrode (now consisting of metallic HVAC tape to further lower material costs) was grounded. Additional material was placed within the mesh acting as a flow control, forcing all compressed air to pass through the plasma region. Additionally, a mask holder was 3D printed to direct plasma flow through the mask and minimize leakage. Figure 2 displays this system via a schematic, a picture and the concurrent circuit diagram. For a more direct comparison between the original and the low-cost system, the same flowmeter was used in both systems, however if implemented the simple 3D-printed stopcock design is able to effectively act as an imprecise flowmeter. Analysis of the electrical output characteristics of this system was performed in the same way as for the system outlined in “DBD plasma reactor for FPR decontamination”.

### Gas composition and characterization

**Figure 3:**
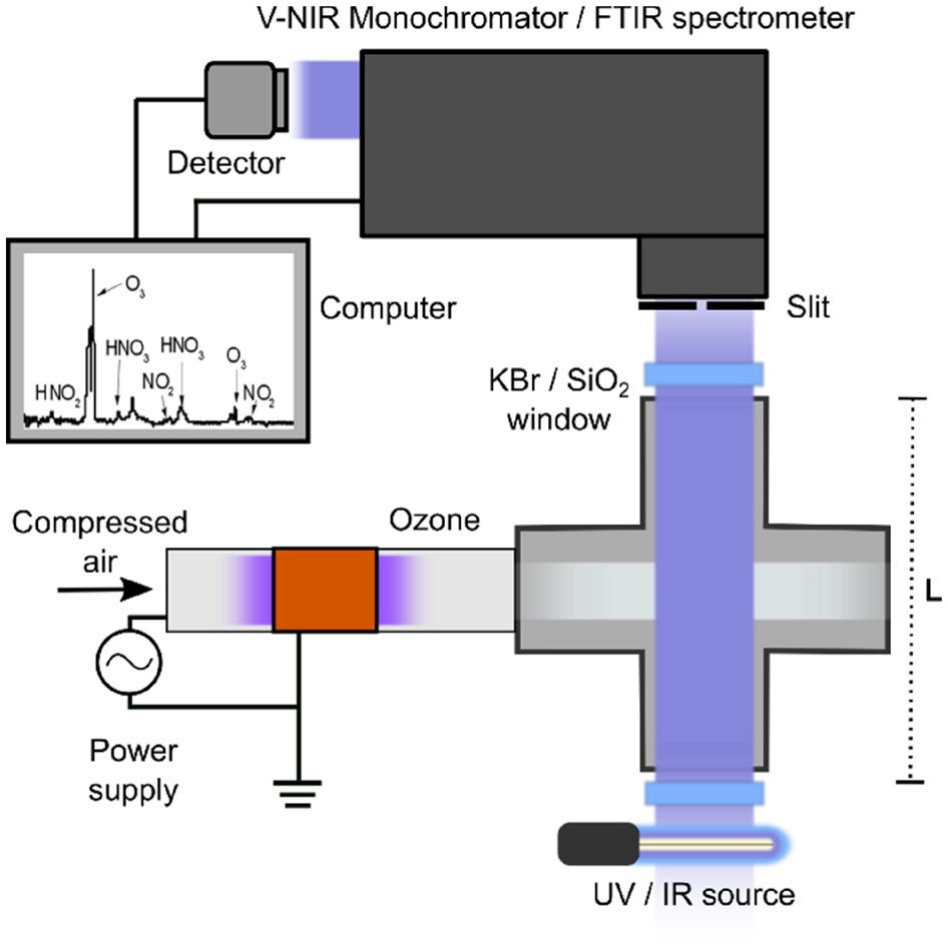
The same system was used to characterize the chemical composition (FTIR spectrometer and IR source) and the ozone concentration (V-INR monochromator and UV lamp) after the plasma discharge.

The power dissipated in the DBD discharge was characterized following the procedure described by Liu et al.^14^. Chemical composition of the gas at the outlet of the plasma discharge was measured via an FTIR spectrometer positioned orthogonally to the plasma stream as shown in Fig. 3. The reactor was located to one side of a stainless-steel cross connector (KF 25) and, perpendicular to the gas flow, an IR source (Newport 80007) was placed in front of a KBr window. The transmitted light through the gas path was collected after another KBr window by a FTIR spectrometer (Nicolet iS50) (from 800 to 4000 cm^−1^; 50 cumulative averages). Absorption spectra were measured as a function of voltage discharge from 0 to 10 kV. For each applied voltage, a background was acquired before striking the plasma and subtracted.

The concentration of ozone after the plasma discharge was carried out by means of UV absorption spectrum with the same configuration used for the chemical composition measurements (see schematic Fig. 3). However, a standard UV lamp (Analytik Jen Pen-Ray 90001201) and V-NR spectrometer (Acton Spectra Pro, Princeton Instruments), connected to a CCD camera, substituted the IR source and the FTIR spectrometer, respectively. The KBr windows were also replaced by SiO_2_ ones to minimize UV light absorption before and after the gas optical path.

The signal intensity at λ = 253 nm, where the peak of the ozone absorption cross section is located^15^, was captured by the CCD camera for different discharge voltages from 0 to 10 kV. In addition, the same signal intensity was recorded at different times (from 0 to 64 min in steps accordingly to the disinfection times) to study the stability of the ozone production. These measurements were used to calculate the ozone concentration by Eq. (1)^16^.

1 $${C}_{oz}\left[g/{m}^{3}\right]=-\frac{{10}^{6}{m}_{oz}}{\sigma LN}\mathrm{ln}\left(\frac{{I}_{oz}}{{I}_{0}}\right)$$

where *I*_*oz*_ and *I*_*o*_ are the intensity of the signal with and without the presence of ozone respectively, *L* is the distance in cm of the light path inside the gas (in this case 10.5 cm), σ is the absorption cross section of ozone at approximately 12 × 10^–18^ cm^2^, m_oz_ is the atomic mass of ozone and *N* is the Avogadro’s number^17^. For consistency with common presentation of ozone concentration as parts per million (ppm), Eq. (1) was multiplied by ${10}^{6}\frac{RT}{{m}_{air}}$, where *R* is the ideal gas constant, *T* is the temperature and *m*_*air*_ is the atomic mass of air. These ozone measurements were then confirmed to within 40 ppm through use of FTIR measurements and the method outlined by Petruci et al.^18^.

### Quantifying bacterial decontamination efficacy

All assays were performed using *E. coli* β10 cells transformed and selected for ampicillin resistance and constitutive expression of super-folding green fluorescent protein (sfGFP). For each biological replicate, 50 mL of LB media (1% tryptone, 0.5% yeast extract and 1% NaCl) were grown to saturation overnight at 37 °C with agitation. Surgical masks were inoculated with 200 µL of culture that were spread on defined 1″ × 1″ hydrophobic (blue side) regions using sterile scoopulas. Masks were allowed to dry for 60 min prior to decontamination.

Inoculated segments were excised from masks using sterile scalpels and placed in sterile 50 mL conical tubes. Masks were suspended in 10 mL sterile water and agitated via pulse vortex for 10 s. Cells were extracted from masks via centrifugation at 2147*g* (4000 RPM) for 10 min. Pelleted cells were resuspended in solution via pulse vortex for 10 s. LB agar plates containing 100 μg/mL ampicillin were inoculated using 200 µL of resuspended cellular solution. Each mask was used to inoculate three agar plates as technical triplicates. Agar plates were incubated at 37 °C for 16 h before green fluorescence imaging using a ChemiDoc MP imaging system (Bio-Rad Laboratories, Hercules, CA).

Decontamination kinetics were modelled as the percent of colony forming units (CFUs) relative to control values. Control masks were placed on the mask holder with the device powered off for 16, 32, or 64 min, while treated masks underwent the same time increment testing with the device powered on. CFUs were counted from fluorescent images using custom MATLAB scripts (MATLAB 2019b; Mathworks, Natick, MA). Control CFUs were calculated as the mean of three biological replicates, and time point measurements are presented as the percent control CFU. Decontamination was modelled as the sum of two exponential decays fit by non-linear least-squares regression in R. Confidence intervals were calculated using a parametric bootstrap with 5000 sample draws.

Efficacies for different decontamination configurations were determined by comparing CFUs on surgical masks after 32 min of treatment. These configurations included passive sterilization (O_3_ flooded box), flow-through DBD, and low-cost flow-through DBD. Negative controls were performed by leaving inoculated masks in the passive O_3_ reactor in the absence of O_3_ for the prescribed amount of time.

A similar experiment was performed using the low-cost flow-through DBD system to decontaminate surgical masks, KN95 FPRs, and cloth facemasks for 32 min. This experiment allowed for the observation of decontamination efficacies on different mask types. Negative controls were treated identically to their treated counterparts but were not exposed to O^3^.

### Quantifying viral decontamination efficacy

Vesicular Stomatitis Virus (VSV), SARS, and COVID-19 are all enveloped, single strand RNA viruses of approximately the same size (60–200 nm)^19,20^ and possess comparable viabilities with alternate sterilization methods^21,22^. VSV was chosen as a functional surrogate to COVID-19 for these similarities, researcher safety concerns, and rapid experimental completion^5^. For our experiments, VSV was replication deficient and expressed Green Fluorescent Protein (VSVΔG*/GFP-G). This allowed for high throughput monitoring of infectivity using flow cytometry to calculate the percentage of cells expressing GFP.

The day prior to face mask inoculation 10,000 BHK cell per well in 50 uL were seeded in flat-bottomed 96 well plates. On the day of inoculation, 200 uL of VSVΔG*G-GFP stock (9 × 10^7^ IU/mL in D10 media) was spread on defined 1″ × 1″ hydrophobic (blue side) regions of a face mask using sterile scoopulas. Masks were allowed to dry for 60 min followed by designated plasma treatment. Inoculated segments were excised from masks using sterile scalpels and placed in sterile 50 mL conical tubes containing 5 mL D10. The mask segment was soaked and mixed for 5 min. 100 μL of media from the conical tube was then add to a well of BHK cells (performed in triplicate). The following day, BHK cells were assessed for GFP positive cells using flow cytometry. Briefly, cells were trypsinized, washed with PBS, then resuspended and analyzed using MACSQuant. Titer (TU, transducing units) was calculated according to the following formula: TU = (P × N / (100 × V)) × TV, where P = % GFP + cells, N = number of cells at time of transduction = 20,000, V = volume of dilution added to each well = 0.1 mL and TV = total volume = 5 mL. TU was zeroed using negative control values. Using the low cost DBD system the same exposure protocol was used for the viral testing as was implemented for the *E. coli*. Namely, control samples at 16, 32, and 64 min undergoing attachment to the facemask holder without activating the device, while treated facemasks underwent the same process with the device on.

### Mask decontamination and assessment of structural integrity

Initial observations on the decontamination process’ effect on overall structural integrity of the fibers of a medical mask was performed with an optical microscope. A medical mask was analyzed with an optical microscope before and after 64 min of ozone treatment (the mask was marked in its center with a sharpie enabling to perform the analysis in a fixed position on the surface of the mask). After observing for alterations in mask structure the question of whether mask filtration efficiency was altered becomes dominant. To test this, Nelson Labs LLC, a 3rd party contractor, was used to perform Sodium Chloride Aerosol Test to determine filtration efficiency on a three sets of identical KN95 masks, with each set consisting of masks that had undergone: 30 min treatment, 60 min treatment, 120 min treatment, and an untreated control. This filtration test is the industrial standard and is performed by generating neutralized polydisperse aerosol particles of NaCl and passing them through the facemask in question. Efficiency is then found by comparing the measured concentration of salt against the challenge concentration, while additional measurements of airflow resistance is also taken. It should be noted that this method provides limited insight into which particle size in the polydisperse aerosol maximizes mask permeability.

## Results and discussion

**Figure 4:**
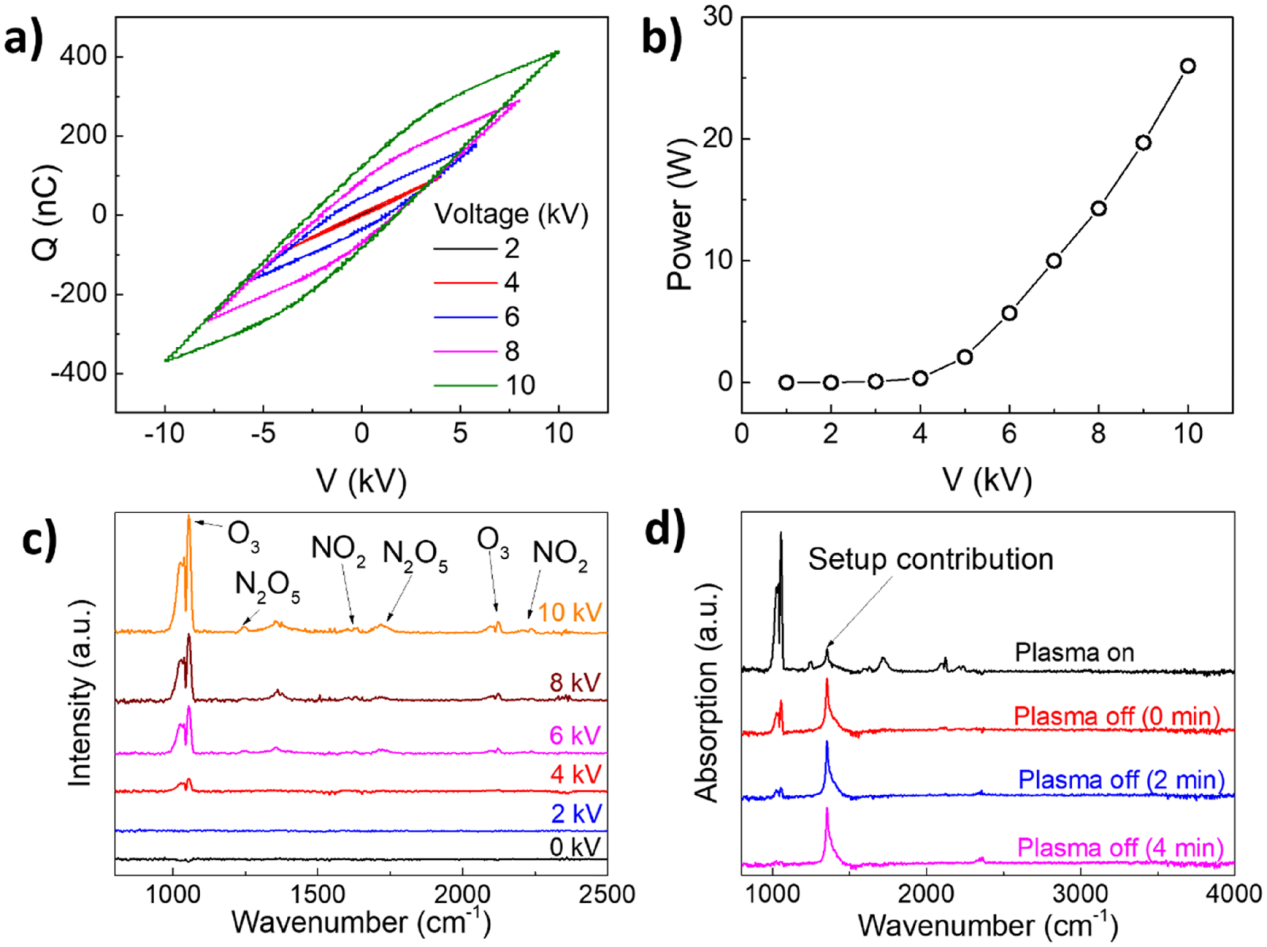
(**a**) Lissajous Figure as a function of applied voltage, (**b**) coupled power and (**c**) FTIR measured downstream of the reacator as a functon of applied voltage. (**d**) FTIR measurement dowstream of the plasma reactor in plasm-on condition, right after switching off the plasa, after 2 min and after 4 min.

To determine dissipated power within the DBD discharge, the applied voltage *V* was measured directly from the output of the power supply, while the current flowing through the electrodes was estimated by measuring the charge *Q* accumulated on a 20 nF measuring capacitor *C*_*M*_ serially connected to the grounded electrode. The Lissajous figure of the DBD discharge was obtained by plotting the measured *Q*–*V* characteristics (see Fig. 4a) and the power dissipated in the discharge was estimated from its area *S* and discharge frequency *f* using Eq. (2) (see Fig. 4b)^14^.

2 $$Power \left(W\right)=f {C}_{M}S$$

The reactor starts coupling a measurable amount of power around 4 kV, linearly increasing with the applied voltage above this threshold (see Fig. 4b). Fourier Transform Infrared Spectroscopic (FTIR) analysis of the gas produced by the DBD discharge corresponds with the appearance of the typical features of ozone (1055 cm^−1^, 1030 cm^−1^, 2098 cm^−1^, and 2121 cm^−1^) with voltages near and above 4 kV (see Fig. 4c)^23^. In air fed DBDs O_3_ production is initiated in the plasma phase by the electron impact dissociation of O_2_ into atomic O (see Eq. (3)). O quickly reacts with O_2_ molecules to form O_3_ via three-body collision (see Eq. (4), being M a third-body collision partner)^24,25^.

3 $${O}_{2}+e\to O+O+e$$

4 $$O+{O}_{2}+M\to {O}_{3}+M$$

Other smaller contributions corresponding to N_x_O_y_ species, such as N_2_O_5_ (1250 cm^−1^ and 1720 cm^−1^) and NO_2_ (1600 cm^−1^ and 1627 cm^−1^), are observed in the spectra^23^. It is worth mentioning that these compounds as well as H_2_O_2_, and reactive molecular radicals are also sterilizing agents produced through the electrical breakdown of air. However, the reactors described in this work do not permit contact of plasma and the facemask which restricts active sterilization being done by longer lived reactive molecules such as O_3._ Finally, we observed a sharp feature around 1360 cm^−1^. The sharp feature was found to be an artifact, attributed to the O_3_-induced oxidation of the KBr windows. To demonstrate this, we acquired a series of FTIR over few minutes after switching off the plasma (Fig. 4d). While the ozone contribution disappears over time, we observe that peak around 1360 cm^−1^ remained unchanged and is hence not related to any gaseous species produced by the plasma discharge.

**Figure 5:**
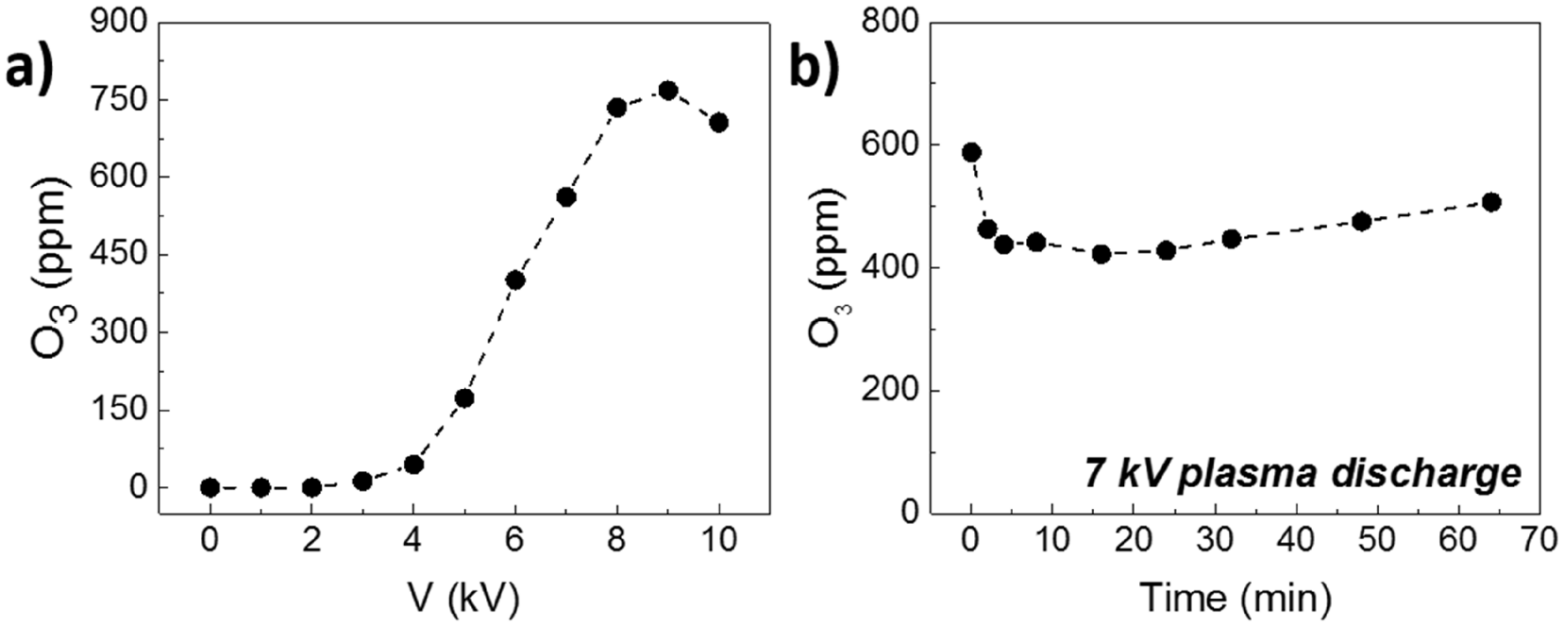
(**a**) Ozone concentration as a function of DBD plasma dishcarge voltage. (**b**) Ozone concentration as a function of time (maximum disinfection time) for 7 kV applied voltage.

The concentration of O_3_ produced by the DBD discharge as a function of the applied voltage was measured via UV absorption spectroscopy, as described previously in “Gas composition and characterization”. In the first set of experiments the plasma was ignited at a given voltage and allowed to stabilize for 4 min before acquiring the measurement. Figure 5a shows a linear increase of the gas concentration above 4 kV, reaching a maximum of 750 ppm approximately at 9 kV and slowly decreasing above this voltage. This effect has been detailed in the work of Yagi et al. on air-fed DBD discharges^23^. As the power consumption of the discharge increases, the ozone production shows a correspondent gradual increase, reaches a maximum and then begins decreasing. This effect is likely due to the production of NO_x_ in the plasma discharge that generates catalytic cycles of O_3_ destruction (see Eqs. (5) and (6)).

5 $${O+NO}_{2}\to NO+{O}_{2}$$

6 $$NO+{O}_{3}\to N{O}_{2}+{O}_{2}$$

For the following decontamination experiments with the DBD system, we fixed the applied voltage at 7 kV as we observed increasing plasma instability above this value. The ozone concentration was sampled over a period of 64 min at 7 kV, to study the overall stability of the DBD production process. As depicted in Fig. 5b, after roughly 4 min, the quantity of produced O_3_ slightly decreases in the first minutes, likely because of the heating of the tube section on which the plasma impinges, but then remains quite stable. On average, the ozone production over 64 min is around 453 ± 27 ppm.

**Figure 6:**
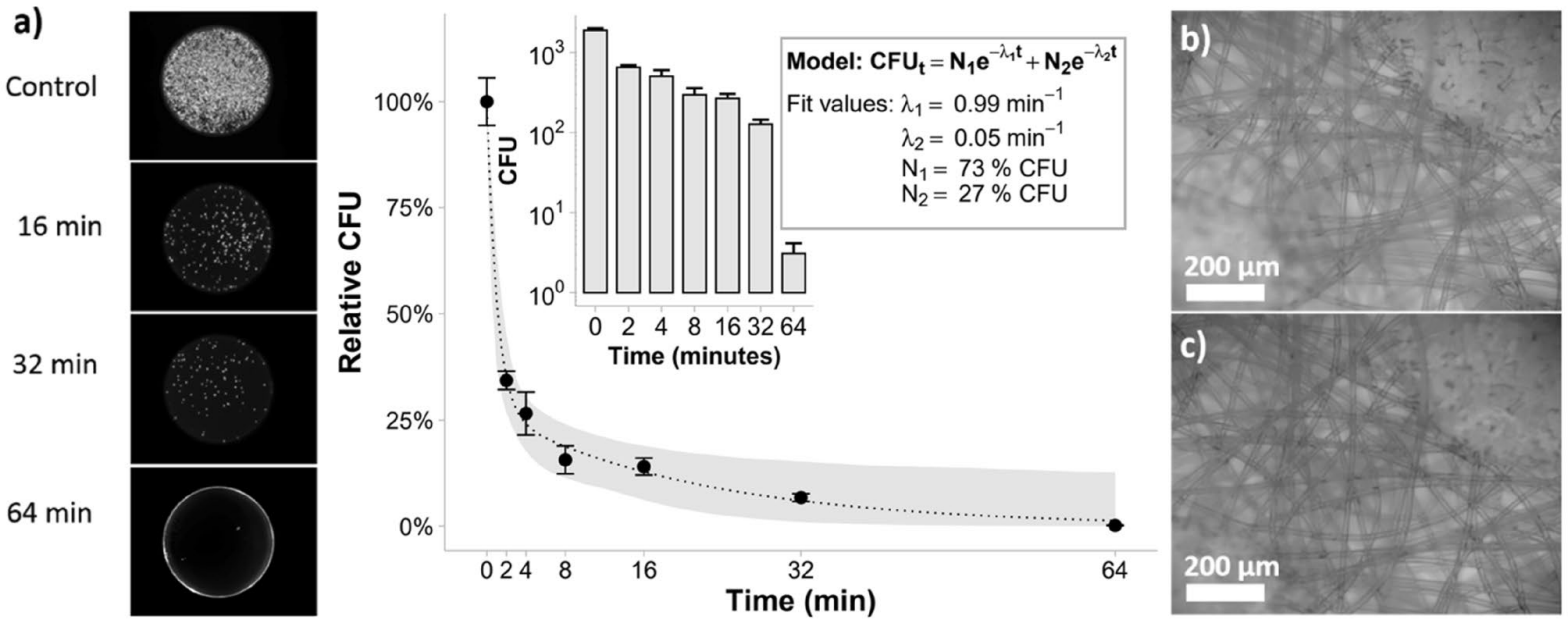
Bacterial decontamination of surgical masks over time. (**a**) Fluorscent images of colony growth on agar after varying decontamination times are portrayed on the left. The image at 64 min (bottom) is overexposed as only a single colony was observed (red). In the scatter plot, each point represents the mean and S.E.M. from three technical replicates that were normalized by their respective control’s mean CFU (mean of control’s CFU was 10^3.42^). Relative CFU were modelled as the sum of two exponential decays. The gray ribbon represents a 95% confidence interval calculated using a parametric bootstrap. After 64 min, we observed a 10^2.78^ reduction in CFU as illustrated in the inlayed plot. (**b**) Optical microscope image of mask before decontamination. (**c**) Optical microscope image of mask after 64 min of O_3_ sterlization. Major discrepancies in strand formation were not obseved.

The decontamination effectiveness was determined using *E. coli* incubated surgical masks (Fig. 6). Between 100,000 and 200,000 CFUs were routinely recovered from control masks. CFUs decreased with increasing exposure time. Notably, the change in CFUs exhibits a biphasic behavior that could be modelled as the sum of two exponential curves. This implies two populations: a fast-dying population with a decay constant λ_1_ (see Fig. 6a) of 0.99 min^−1^ and a mean lifetime of approximately 1 min, and a slow-dying population with a decay constant λ_2_ (see Fig. 6a) of 0.05 min^−1^ and a mean lifetime of approximately 20 min. Across all experiments, we observed a 3:1 ratio (N_1_:N_2_, see Fig. 6a) between fast and slow-dying populations and we speculate that the slow-dying population has reduced ozone exposure due to fouling from the saturated bacterial culture. This set of experiments indicated that most bacteria are quickly killed over the first few minutes of the disinfection process, and a bacterial reduction greater than three orders of magnitude is achieved within 64 min (Fig. 6a). Finally, we performed an analysis of the morphology of the medical mask before and after treatment, to assess any possible structural damage induced by the O_3_ treatment (Fig. 6b,c). There was no observable variation of the fiber structure, consistent with the near room temperature operating conditions this method utilizes.

**Figure 7:**
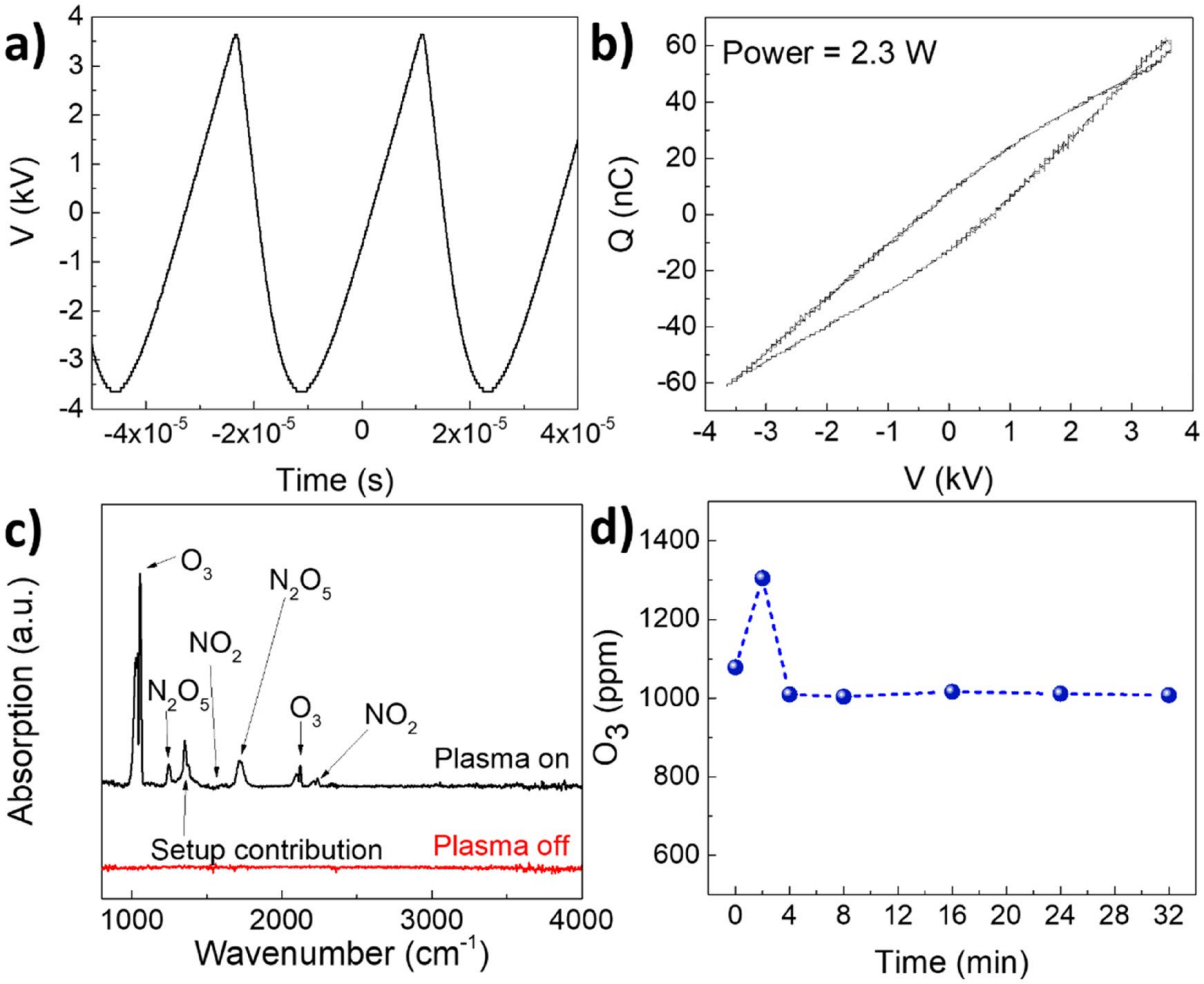
(**a**) Voltage signal produced by the power supply of the plasma globe. (**b**) Lissajous Figure of the plasma globe reactor. (**c**) FTIR analysis of the gas composition produced by the Plasma Globe Reactor and (**d**) corresponding O_3_ concentration produced by the plasma globe reactor as a function of time. An average of 1010 ± 5 ppm along the stability period (4 to 32 min).

After verifying the effectiveness of the O_3_ based decontamination in removing bacteria from surgical masks, we explored the characteristics of the “portable-version” of the decontamination apparatus built using the plasma globe toy. The power supply feeds the discharge electrode with a sawtooth signal with frequency equal to roughly 30 kHz and a peak-to-peak amplitude of 5.6 kV, corresponding to a discharge power of 2 W (Fig. 7a,b). Surprisingly, in this configuration we observe an extremely stable O_3_ production with average concentration, in the order of 1000 ppm (Fig. 7c,d).

**Figure 8:**
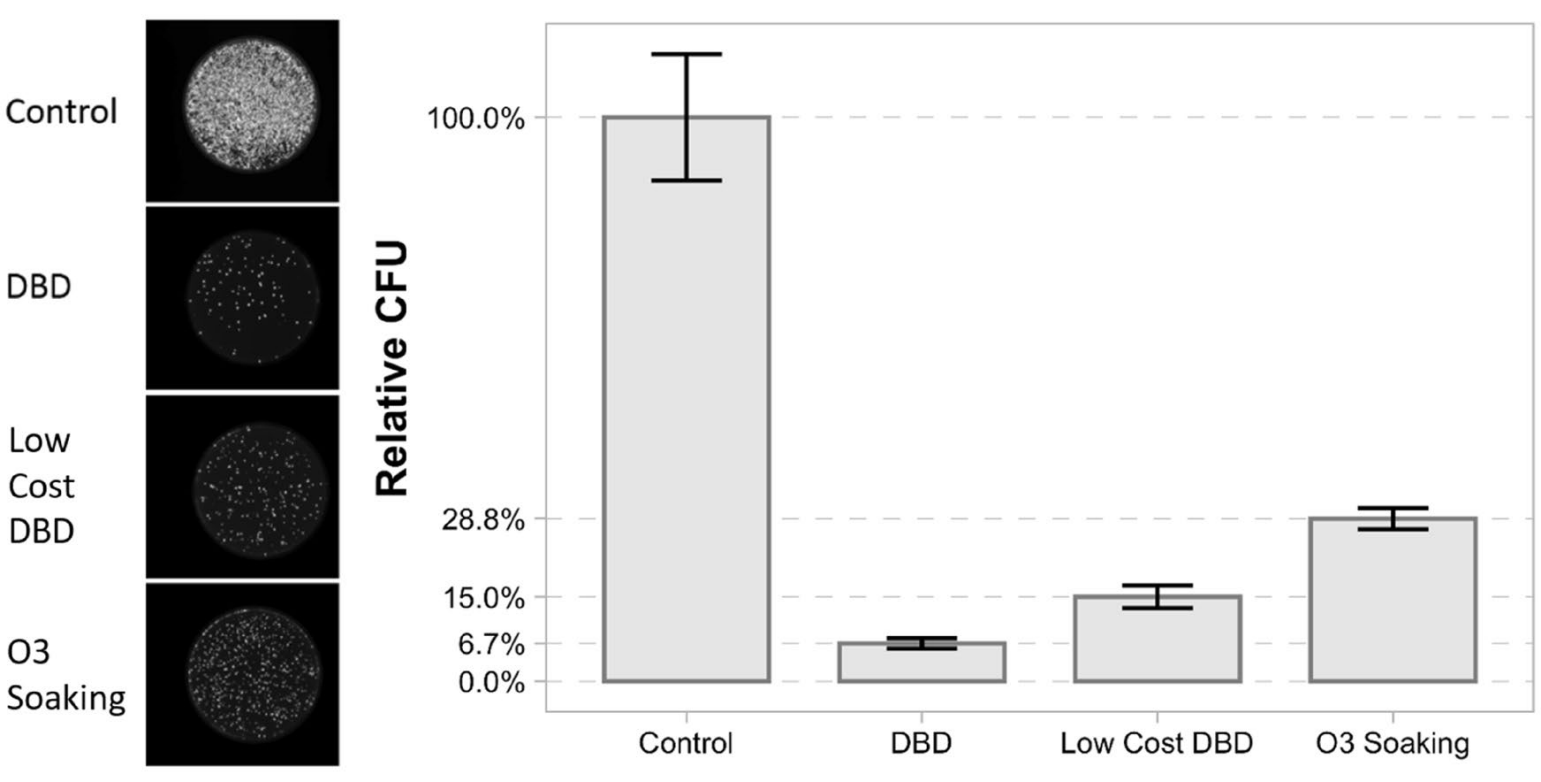
Decontamination efficacy using different configurations. The left inset shows fluorescent images of colony growth on agar after 32 min of treatment using different configurations. Right graph shows CFU values of different configurations relative to the negative control.

Finally, the efficacy of the low cost DBD device was measured for 32 min treatment time and compared with the one of the DBD and the more widespread configuration where masks are simply placed in a box that is then flooded with O_3_ and left to soak (for this case the mask was simply unplugged from the mask holder and placed in the closed 72 L plastic box containing the plasma reactor; the DBD was operated at 7 kV with compressed air flow rate of 10 slm). Correspondingly, we observe a 429% improvement in the decontamination efficacy with respect to the standard configuration. Results are summarized in Fig. 8.

**Figure 9:**
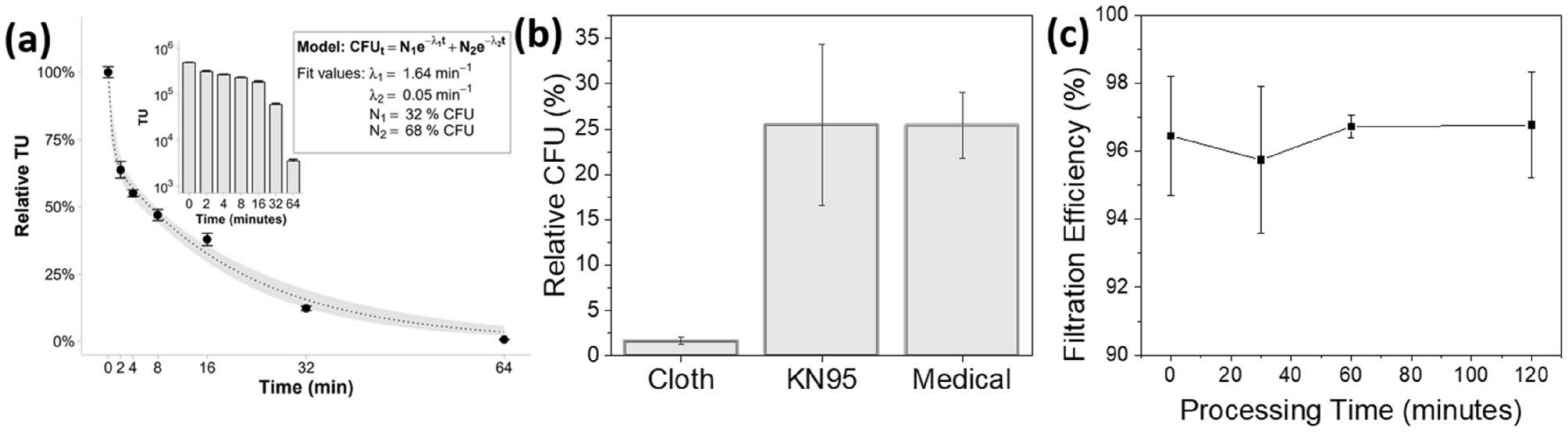
VSV treatment results (**a**) VSV decontamination of surgical masks over time. Each point represents the mean and S.E.M. from three biological replicates that were normalized by the control’s mean TU (mean of control TU was 10^5.68^). Relative TU were modelled as the sum of two exponential decays. The gray ribbon represents a 95% confidence interval calculated using a parametric bootstrap. After 64 min, we observed a 10^2.13^ reduction in TU as illustrated in the inlayed plot. (**b**) Decontamination efficacy after 30 min using low-cost system on different mask types. (**c**) Filtration efficiency of KN95 masks as a function of treatment time. No significant changes in filtration was observed.

These results provide conclusive evidence that the forced ozone disinfectant method is effective on bacterial pathogens, does not induce structural damage to mask fibers, and can be implemented into a portable configuration using low-cost components. Next, we confirm the decontamination efficacy of this scheme on a viral pathogen physically comparable to those prompting the use of facemasks (eg. SARS, MERS, COVID-19). For this, VSV with GFP was used and analyzed via flow cytometry. Utilizing the low-cost setup, we treated the same masks for the same time intervals as done with the *E. coli* described in “Quantifying viral decontamination efficacy”. Similarly to the *E. coli*, we regress on VSV population over time using a two population exponential decay model. Similarly to *E. coli,* we observe that approximately 68% of the population dies quickly with a 3 s half-life and the remainder die slowly with a near 100 s half-life. Also similar to *E. coli*, decontamination for 64 min resulted in a pathogen reduction of greater than two orders of magnitude (shown in Fig. 9a).

Next, we demonstrate that this method is both functional and non-destructive for different mask types. To accomplish this, a side-by-side comparison between medical masks, thick cotton masks, and KN95 masks was performed using the same pathogen and processing conditions as for the *E. coli* time trial, performed with the low-cost system. Each mask was sterilized for 30 min and upon processing has shown the more absorbent masks to have sterilized more rapidly (see Fig. 9b). We believe this to be a result of the cotton fibers having a lower surface tension allowing the viral load to cover more area and as a result be more accessible to the ozone as it flowed through the mask. Finally, this technology does not adversely affect the filtration efficiency of the masks. To test this a series of KN95 masks were exposed to the ozone-rich air stream produced via low-temperature plasma then underwent blind 3^rd^ party NaCl filtration testing at Nelson Labs. The obtained results show no impact on mask filtration efficiency, as shown in Fig. 9c, with the filtration efficiency exceeding the KN95 standard of 95% for all the treatment durations.

## Conclusion

We have demonstrated that the efficiency of an ozone decontamination system for facepiece respirators can be dramatically increased by careful design of the reactor configurations. Specifically, a flow-through configuration where the ozone is passed directly through the porous fiber structure of the mask demonstrated superior decontamination kinetics with respect to the standard approach of an ozone chamber. This method has proven effective against both viral and bacterial pathogens causing a reduction of active pathogens by a minimum of two orders of magnitude within the first hour of processing. Treatment has also proven to be non-destructive to the mask’s physical structure and does not reduce filtration efficiency over time. Finally, this method has been demonstrated to be effective when reconstructed for use as a portable single mask treatment device using low-cost commercially available components (a plasma ball toy, a plastic box, a quartz tube, some steel mesh, HVAC tape, and a few 3D printed parts). The combination of efficacy, low-cost and potential portability make this approach a viable option to (a) extend the lifetime of facepiece respirators and enable their safe reutilization, (b) reduce waste and (c) help the healthcare system face periods of crisis, such as the one recently witnessed at the onset of the COVID pandemic, in which shortages of personal protective equipment put professionals at risk.
